# The Treatment of Fractures from a Common-Sense Point of View

**Published:** 1907-12-14

**Authors:** 


### Points in Surgery.

### THE TREATMENT OF FRACTURES FROM A COMMON-SENSE POINT OF VIEW.

### XVI.—POTT'S FRACTURE.

The term Pott's fracture is often loosely used to describe any fracture of the bones of the leg in the neighbourhood of the ankle joint. Strictly the name should be confined to fractures of the lower end of the fibula associated with outward displacement of the foot and either (i) fracture of the internal malleolus of the tibia or (ii) rupture of the internal lateral ligament of the ankle joint. This condition is nearly always the result of indirect violence, the patient slipping and twisting the foot outwards. The fracture probably occurs secondarily as the result of the strain produced by the extreme eversion. The fibula gives way at its weakest point, which is about three inches above the external malleolus. Last of all the bone or ligament on the inner side of the foot is unable to withstand the strain put upon it, and it gives way also. In a certain percentage of these fractures the condition is complicated by the presence of a skin wound over the internal malleolus. When the internal lateral ligament is ruptured it is the apex of the malleolus that does the damage, but when the malleolus itself is fractured, it is generally the sharp projecting edge of its base that implicates the skin. The fracture thus becomes a compound one; and this may happen at the time of the accident, or later from subsequent ulceration or gangrene of the skin.

#### Results.

It is a matter of no small difficulty to obtain a really good result after Pott's fracture. The great trouble is not in reducing the deformity, but in retaining the fragments in position after reduction. If anything, the deformity should be somewhat over-reduced. It is a sound general rule which says that the fragments are in satisfactory position if the following three points are in the same straight line, the inner side of the ball of the great toe, the internal malleolus, and the inner border of the patella.

In an ordinary case—that is, one in which the skin is not implicated—it is best to give an anæsthetic and reduce the displacement, and to proceed at once to put the limb up in a Bavarian plaster of Paris splint while the patient is still anæsthetised. After reduction and before putting on the splint it is well to see that movement at the ankle joint is free and is not resisted by any displaced tendons. The advantage of immediately putting on a plaster of Paris splint is that the limb can be held in the desired position while the plaster dries, and there is no possibility of a recurrence of the deformity. Of course, the plaster-splint must be opened in twenty-four hours, or even less if any effusion makes its appearance and so hinders the venous return from the foot. It can be seen if this is about to happen by watching the condition of the toes. If there is any pressure under the splint they become swollen and dusky in colour. It is also extremely essential to begin passive movement at the earliest possible moment in these cases, because the joint is nearly always involved, and limitation of movement at the ankle is a common sequela.

But if the fracture is a compound one when first seen, or if there is any doubt as to whether the vitality of the skin over the malleolus is impaired or not, it is obvious that some form of apparatus must be employed which will admit of the skin being seen and the wound, if there is one, dressed daily. In the writer's experience nothing is so good as the back splint and two side splints, as advised for fractures of the shaft of the tibia. It is true that inversion of the foot can be more surely obtained with a Dupuytren's splint. This is a splint similar to Liston's long splint, but shorter. It is applied to the inner side of the limb. It is thickly padded as far down as the base of the internal malleolus but not below this; so that the foot can be strongly inverted by bandaging it to the splint; but it has the disadvantage that it does not counteract the tendency of the whole foot to fall backwards owing to its weight. Syme's horseshoe splint on the other hand prevents backward displacement of the foot, but does not produce sufficient inversion. The back and side splints, however, answer the purpose well; backward displacement cannot occur, and inversion can be insured by keeping a thick pad between the foot and the outer side splint.

#### After Treatment.

The splint can be discarded after three or four weeks, but it is important not to let the patient attempt to walk for seven or eight weeks. If he does this before union is sufficiently advanced the weight of the body may cause the foot to assume a valgus position, which is incurable; so that his condition is practically the same as that of a patient with a Pott's fracture, in whom the deformity has either not been corrected at all or has been insufficiently corrected. It may be possible in such cases to benefit the condition by an open operation in which the original condition is reproduced and treatment started again *de novo*.

It is an open question whether it is advisable to wire or peg the fractured malleolus to the shaft in these cases. It seems reasonable to do so in compound fractures where the bones are exposed, on the ground that the subsequent treatment will be easier, because there will be less tendency for the displacement to recur. But if the fracture is a simple one, it seems sounder treatment to try every other means of getting the bones to unite in a proper position before inserting what is, to all intents and purposes, a foreign body into the bone.

